# Advances in Diagnosis and Management of Hemodynamic Instability in Neonatal Shock

**DOI:** 10.3389/fped.2018.00002

**Published:** 2018-01-19

**Authors:** Yogen Singh, Anup C. Katheria, Farha Vora

**Affiliations:** ^1^Department of Pediatric Cardiology and Neonatal Medicine, Cambridge University Hospitals NHS Foundation Trust, Cambridge, United Kingdom; ^2^University of Cambridge Clinical School of Medicine, Cambridge, United Kingdom; ^3^Department of Neonatology, Sharp Mary Birch Hospital for Women & Newborns, San Diego, CA, United States; ^4^Department of Neonatology, Loma Linda University School of Medicine, Loma Linda, CA, United States

**Keywords:** neonatal shock, functional echocardiography, cardiac output, tissue perfusion, hemodynamic

## Abstract

Shock in newborn infants has unique etiopathologic origins that require careful assessment to direct specific interventions. Early diagnosis is key to successful management. Unlike adults and pediatric patients, shock in newborn infants is often recognized in the uncompensated phase by the presence of hypotension, which may be too late. The routine methods of evaluation used in the adult and pediatric population are often invasive and less feasible. We aim to discuss the pathophysiology in shock in newborn infants, including the transitional changes at birth and unique features that contribute to the challenges in early identification. Special emphasis has been placed on bedside focused echocardiography/focused cardiac ultrasound, which can be used as an additional tool for early, neonatologist driven, ongoing evaluation and management. An approach to goal oriented management of shock has been described and how bed side functional echocardiography can help in making a logical choice of intervention (fluid therapy, inotropic therapy or vasopressor therapy) in infants with shock.

## Background

The key to the management of shock in the newborn period is early identification and determination of etiology to provide appropriate care. American College of Critical Care Medicine (ACCM) published clinical guidelines and practice parameters to promote “best practices” and to improve patient outcomes in pediatric and neonatal septic shock in 2002, with a subsequent update in 2007 (1). In addition to emphasizing early recognition and instituting goal oriented, time sensitive interventions, these guidelines also support the use of hemodynamic parameters, specifically central venous oxygen saturation and cardiac index, in ongoing management of shock in the neonatal intensive care unit (NICU).

Despite widespread dissemination of such guidelines, management of neonatal shock continues to rely on traditional methods of monitoring and management. We aim to discuss the unique pathophysiology associated with shock in newborns, with a focus on the very low birth weight (VLBW) infants, in addition to discussing newer modalities for hemodynamic monitoring, and the role of bedside functional echocardiography in management of neonatal shock.

## Definition of Shock

Shock is a pathophysiologic state characterized by an imbalance between oxygen delivery and oxygen demand in the tissues leading to tissue hypoxia. The initial compensated phase is characterized by neuroendocrine compensatory mechanisms with increased tissue oxygen extraction, leading to maintenance of blood pressure (BP) in the normal range. The blood flow and oxygen supply to vital organs are maintained at the expense of non-vital organs. The compensated phase may have additional signs such as tachycardia, prolonged capillary refill time (CRT) and decreased urine output. In adults, these features are commonly seen early in compensated shock. However, these features may be missed in neonates, in whom shock is mostly recognized in the uncompensated phase. This is in part due to the lack of data on normal BP ranges that would ensure adequate organ perfusion in the premature infant. The uncompensated phase of shock is characterized by a decrease in vital and non-vital organ perfusion, which may be evident by the development of lactic acidosis. This will eventually lead to cellular disruption with irreversible damage, clinically characterized by multiorgan failure and death.

## Pathophysiology of Neonatal Shock

Myocardial dysfunction, abnormal peripheral vasoregulation and hypovolemia leading to decreased delivery of oxygen and nutrients to tissues are often the primary sources of neonatal shock. This is often complicated by relative adrenal insufficiency often seen in the premature infant. The causes and types of neonatal shock are described in Table 1.

**Table 1:** Showing mechanisms of neonatal shock leading to poor tissue perfusion.

Mechanism for poor tissue perfusion	Types of neonatal shock	Causes of shock
Abnormalities within the vascular beds	Distributive shock	Sepsis, endothelial injury, and vasodilators
Defects of the pump	Cardiogenic shock	Congenital heart disease, heart failure, arrhythmia, cardiomyopathy, and post-cardiac surgery/post-patency of the ductus arteriosus ligation
Inadequate blood volume	Hypovolemic shock	Blood loss from infants or placenta around birth of infants
Flow restriction	Obstructive shock	Cardiac tamponade, pneumothorax, high pulmonary vascular resistance restricting blood flow such as in persistent pulmonary hypertension of the newborn, pulmonary hypertension
Inadequate oxygen-releasing capacity	Dissociative shock	Methemoglobinemia and severe anemia

The neonatal myocardium has fewer contractile elements compared with older children and adults (2). In particular, immature myocardium has a higher basal contractile state and has higher sensitivity to changes in afterload (3). This is especially important in the context of the removal of placenta which is low vascular resistance state and transition to the higher vascular resistance state at birth. This is further evidenced by the low superior vena cava (SVC) flow seen in a large proportion of infants in the first 6–12 h of life (4). Other features such as higher water content, greater surface-to-volume ratio, immature sarcoplasmic reticulum and reliance on extracellular calcium stores further render neonatal myocardium incapable of adapting adequately to the changes at birth. This can be further complicated by factors leading to fetal hypoxia and perinatal depression leading to metabolic acidosis and poor myocardial function (5).

This is distinct to the myocardial dysfunction beyond the transitional period when immature myocardium may have a lesser role to play. Hemodynamically significant PDA (hsPDA) is a common cause of hypotension in VLBW infants. The presence of an hsPDA with resultant decrease in diastolic BPs can also theoretically affect the perfusion of the myocardium, which primarily takes place during diastole. However, studies show no significant change in contractility with an hsPDA (6, 7). On the contrary, there may be an initial increase in the left ventricular output secondary to an increase in left ventricular preload in the presence of left to right shunt. The failure of such compensatory mechanisms in the infant may, however, ultimately lead to systemic hypoperfusion. Following ligation, the acute changes in the myocyte fiber length due to the change in left ventricular preload can also affect myocardial contractility before the myocardium adapts to the new loading condition. Finally, any condition leading to asphyxia and/or inadequate perfusion to the myocardium can further compromise the function. Examples in the NICU may include structural heart conditions, arrhythmia, or cardiomyopathies.

The vascular smooth muscle tone and its complex regulation play a key role in pathogenesis of neonatal shock. A balance of the vasodilating and vasoconstricting forces regulates the tone. These factors may involve autocrine, endocrine, paracrine, and neuronal factors. Commonly described factors include vasopressin, nitric oxide, eicosanoids, catecholamines, and endothelin (8–12). A key effect may involve alteration in cytosolic calcium concentration. The role of adenosine triphosphate dependent K channels in the vascular smooth muscle tone has been recently studied (13). The immaturity of the autonomic nervous system of infant also affects the circulatory function and vascular tone (14, 15).

Unlike in the pediatric or adult population, hypovolemia is not a very commonly encountered etiology of shock in the first few days of life. Causes of hypovolemia in newborns would include history of *in utero* blood loss such as with maternal abruption, fetomaternal or fetoplacental hemorrhage or tight nuchal cord. Postnatally, acute blood loss may be associated with gut perforation following necrotizing enterocolitis, sub-galeal bleeding or intracranial hemorrhage. In addition to this, relative hypovolemia can be seen with capillary leak and vasodilatory shock in severe sepsis.

Pathophysiology of shock in newborns is unique since it is associated with physiologic transition from fetal circulation to neonatal circulation at birth. Suprasystemic pulmonary vascular resistance (PVR) in the prenatal period may remain elevated, especially in the presence of ongoing hypoxia and acidosis from sepsis, leading to persistent pulmonary hypertension of the newborn (PPHN). The latter contributes to right ventricular failure, and as such may need therapies directed to decrease right sided pressures. In addition to PPHN, newborn shock may be associated with closure of ductus arteriosus in a ductal dependent congenital heart lesion, as such requiring prostaglandin infusion to open and maintain patency of the ductus arteriosus (PDA).

In addition, there is plenty of evidence suggesting low cortisol levels in sick term, late preterm, and preterm infants (16–19). Both adrenal insufficiency and decreased vascular responsiveness to catecholamines can contribute to vasopressor resistant shock (20).Low dose steroids have been found to improve cardiovascular status in infants with vasopressor resistant shock, further supporting the role of relative adrenal insufficiency (21–23).

## Assessment of Cardiac Output (CO) and Tissue Perfusion

Adequacy of the systemic and peripheral blood flow and thus oxygen delivery to the tissues can be measured by monitoring BP, CO, and/or systemic vascular resistance (SVR).

Direct yet invasive measures of cardiovascular function such as CO measurement *via* thermodilution or pulse induced contour cardiac output, pulmonary wedge pressure, or central venous pressure providing accurate assessment in adults or older children may be impractical in the premature infant. In addition to the difficulty associated with intracardiac shunt placement in VLBW infants, the dye dilution and thermodilution methods may not be accurate due to the presence of intracardiac and ductal shunts.

**Figure 1:**
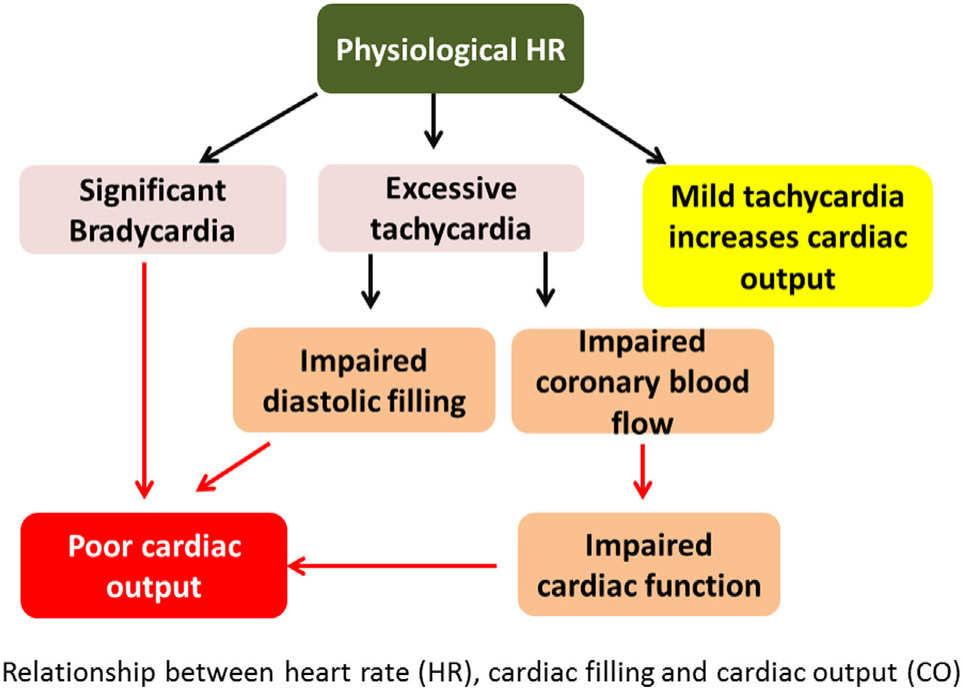
Relationship between heart rate (HR), cardiac filling, and cardiac output (CO). Excessive tachycardia may decrease CO by decreasing preload and hence stroke volume. It may also impair cardiac function from decreased coronary perfusion in shortened diastole.

It is known that BP = CO × SVR. Both BP and CO can be measured. SVR is a derived value from the above equation. CO = heart rate (HR) × stroke volume (SV). SV depends on preload, myocardial contractility, and afterload conditions. The relationship between HR, cardiac filling, and CO has been shown in Figure 1.

Measurement of myocardial contractility using load dependent measures such as fractional shortening (FS) can be affected by the right ventricular dominance characteristic of fetal circulation. Appropriate assessment of myocardial activity requires measurement of load independent measures such as relation between velocity of circumferential fiber shortening and left ventricle (LV) wall stress indices (24).

Blood pressure monitoring, preferably measured invasively can offer continuous real time assessment of the CO (25). However, lack of consensus definition of hypotension in the neonate continues to be a major hindrance to the use of BP as an adequate measure for such an assessment (26). BP may be affected by demographic factors such as birth weight, gestational age, and postnatal age; and coexistent clinical factors such as antenatal steroids, PDA, level of respiratory support, or therapeutic hypothermia. BP is directly affected by SVR, which in turn is regulated by multiple factors including drugs, sepsis, temperature, and hormonal changes. Hence, it may not be the best measure of tissue perfusion. In addition, presence of intra-atrial and ductal shunting may not allow the assumption that ventricular output is an accurate measure of systemic blood flow (27, 28). Mean BP value less than the gestational age in weeks is often considered adequate in the first few days of life (29–31), but this is rather simplistic since thresholds may vary between different patients, and at different time points in the same patient. Hence, attention must be paid to additional measures of perfusion.

Arguably, flow is a better indicator of perfusion than the BP that drives the flow to the organs. However, flow measures such as LV and right ventricle (RV) output may not be accurately depictive of organ blood flow in VLBW infants due to the presence of above mentioned shunts in the transitional period. SVC flow may be used as a valid indicator of cerebral blood flow (CBF) (32–34). In fact, low SVC flow has been shown to be strongly associated with subsequent IVH or neurodevelopmental impairment (33–35). However, recent studies have shown lack of sensitivity of SVC flow in predicting IVH (36). There remain concerns regarding significant intraobserver and interobserver variability in assessing SVC flow and hence its repeatability. This makes it difficult to use in clinical practice, although trend can be useful. Adequate SVC flow is used as one of therapeutic endpoints per the ACCM guidelines (1).

The relation between mean BP and systemic blood flow is a complex one in a VLBW infant, especially in the first few days of life. Autoregulation ensures adequate perfusion to vital organs in states of hypoperfusion. However, cerebral autoregulation may be lacking in the VLBW transitionally at birth or during period of illness. 30 mm Hg has been proposed as the cutoff for mean BP below which cerebral perfusion may not be adequate; however, this relation may not be entirely accurate given differing findings from various studies (37–39).

Other indirect clinical measures of cardiovascular function include CRT, urine output, HR, and presence of lactic acidosis. A combination of such measures rather than individual assessments may offer increased specificity for detecting low flow states (40).

Central venous pressure approximates right atrial pressure and can give valuable information regarding the preloading conditions and in assessing response to volume in critically ill patients. Normal numbers have been described for term and preterm infants, with special emphasis on the trend pattern. However, feasibility in the newborn infants remains questionable owing to the invasive nature of the procedure (41–43).

Mixed venous oxygen saturation (SvO_2_) is considered as the balance between oxygen demand and delivery and has been used as a determinant for tissue hypoxia. It is one of the key targets (in addition to the determinants for preload and contractility) for goal directed management in severe sepsis and septic shock (44). Values both below normal and supranormal have been associated with poor outcomes (45, 46). Normal values for central venous oxygen saturation (ScVO_2_) in preterm infants have also been described, but widespread use is limited by the invasive nature of the procedure along with the effect of persisting fetal shunts in the newborn period (47). ScVO_2_ is another therapeutic end point as suggested by ACCM guidelines.

Arterial venous (A-V) oxygen difference is the difference between the oxygen content of the arterial and venous blood. The normal numbers would be less than 5 ml/100 ml of blood or 25%. In low output states, A-V extraction increases, decreasing the mixed venous saturation, and hence increasing the difference. In distributive shock, there is decrease in the oxygen extraction, leading to higher mixed venous saturation and hence a narrowed difference. This measure offers an excellent estimate of tissue oxygen delivery, however, limited again by the invasiveness of the procedure.

Electrical cardiometry (Aesculon; Cardiotronic; La Jolla, CA, USA) allows for assessment of CO by measuring the changes in thoracic electrical bioimpedance caused by the cardiac cycle. It is non-invasive, easy to apply, offers continuous assessment and has recently been validated against invasive methods of CO measurements in hemodynamically stable newborns. However, more data are needed for validation in neonates with hemodynamic compromise before its widespread clinical applicability (48, 49).In addition, sicker infants on significant ventilator support (i.e., high frequency oscillation) may have poor correlation compared with echocardiography derived COs (50).

Near infrared spectroscopy (NIRS) has been evaluated as a tool to assess cerebral and peripheral oxygenation and oxygen extraction. The benefit of this modality is the availability of continuous measurement. Among other measures, NIRS can provide values for cerebral oxygenation (rScO_2_) and cerebral fractional tissue oxygen extraction. Reference values with comparison with other modalities have now been published, but greater clinical applicability rests with trend monitoring rather than absolute numbers (51–53). There remains paucity of data in preterm infants and even in term infants there remain concerns regarding when intervention should be based primarily based on rScO_2_. Moreover, NIRS values remain quite non-specific and hence unreliable for the abdominal organs. The significance of studying NIRS of the kidney is being evaluated, and it may be used as a reflection of perfusion in future. In practice, with the available data NIRS can be useful for cerebral oxygenation monitoring in term infants but we need more convincing data for its use in preterm infants and for gut perfusion before it can be incorporated in clinical practice guidelines. Cerebral perfusion measured by NIRS can give a good idea about the adequacy of cerebral perfusion. A recently completed multicentre trial comparing blinded versus unblinded NIRS did demonstrate a reduction in cerebral hyperoxia and hypoxia with a trend toward lower mortality in the unblended NIRS group (54).

The use of Visible Light Technology (VLS, T-Stat; Spectros, Portola Valley, CA, USA) has been described for continuous assessment of capillary oxygen saturation in various organs (55, 56).Unlike pulse oximetry, VLS measurements are not affected by conditions of local ischemia, lack of pulsatile flow, vasoconstriction, or hypothermia. More data are needed in newborns with hemodynamic compromise to validate the correlation between the changes in SVR and the hemoximetry findings with T-Stat.

The plethysmographic signal of pulse oximeter can be used to calculate ratio of the pulsatile and non-pulsatile components, described as Perfusion Index. This has been recently studied and is found to be reasonably predictive of low flow states, including patent ductus arteriosus (57–59). Reference values still need to be established in the preterm infant in whom PI is significantly affected by the transitional circulation, taking up to 72 h for the values to stabilize (59). Hence, limiting the utility of PI in preterm infants during early life.

Functional cardiac MRI has been recently evaluated as an additional feasible tool to evaluate cardiac hemodynamics, especially PDA. At this time it is deemed to be an insightful research tool while awaiting more studies (60, 61). Role of bedside echocardiography, especially Doppler ultrasonography is further discussed in one of the following sections. All the parameters used for assessment of neonatal shock are summarized in Table 2.

**Table 2:** List of the parameters used for assessment of neonatal shock.

Conventional parameters (commonly used in standard practice)	Capillary refill time Urine output Heart rate Blood pressure Presence of lactic acidosis Central venous pressure Mixed venous saturation Arterio venous oxygen difference
New parameters (now being used in clinical practice)	Functional echocardiography Near infrared spectroscopy
Novel parameters (research tools at this time, not being used in clinical practice)	Electrical cardiometry Visible light spectroscopy Perfusion Index Functional cardiac MRI

## Management of Neonatal Shock—Clinical Essentials in Management of Shock

**Figure 2:**
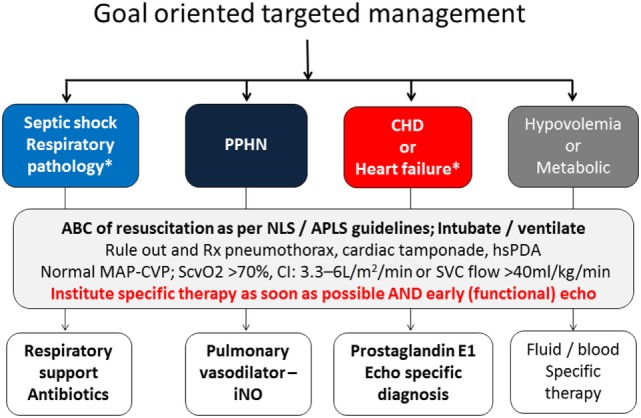
Goal oriented targeted management and role of echocardiography in instituting specific intervention.

Key to the management is early recognition and identifying the underlying pathophysiology of shock. The earlier findings include pallor, poor feeding, tachycardia, tachypnea, and temperature instability. As discussed earlier, hypotension is a late finding in neonatal shock. In addition, other late features may include weak peripheral pulses, low ScvO_2_, signs of decreased peripheral perfusion such as acidosis, elevated lactate. In spite of being a late finding, hypotension is the most commonly used determinant of decreased perfusion in NICU given the ease of monitoring. The clinician must keep a keen eye on the other signs of symptoms discussed earlier before the “ischemic threshold” is reached for low BP. The other traditionally used clinical parameters such as clinical assessment, HR, CRT, urine output, and serum lactate are also proxy indirect markers of cardiovascular well-being. Early bedside focused echocardiography, described by some as focused cardiac ultrasound, can help in early identification of underlying pathophysiology and targeting specific therapy. The aim of focused echocardiography is not to rule out congenital heart defect but to gain physiological information which can help in delivering goal oriented time specific intervention. Interventions should be based after carefully considering the underlying pathophysiology (Figure 2). In addition to the modalities discussed earlier, functional echocardiography and NIRS can give the neonatologist a unique skill to evaluate reliable measures of organ perfusion and monitor changes following intervention. This is further discussed in the next section.

The ACCM guidelines have established goals and therapeutic end points for the management of shock in both delivery room and subsequently in the NICU. The therapeutic end points in the first hour of resuscitation include CRT ≤ 2 s, normal and equal central and peripheral pulses, warm extremities, urine output >1 ml/kg/h, normal mental status, normal BP for age, normal glucose, and calcium concentrations (62).

Along with consideration of interventions to increase BP, attention should be paid to conditions contributing to hypoperfusion. These may include but not limited to patency of ductus arteriosus, sepsis, excessive mean airway pressure, pneumothorax, and adrenal insufficiency. These should be addressed accordingly. The common interventions used in NICU to improve BP include use of inotropes/vasopressors, volume resuscitation, and steroid administration.

There is no clear correlation between blood volume and BP in neonate (63). Hypovolemia is rarely the primary cause of hypotension in the VLBW infant in the first few days of life, unless there is clear history of perinatal blood loss. Indeed, studies have shown that dopamine is more effective in correcting hypotension compared with fluid resuscitation in the immediate postnatal period (64). In addition, excessive fluid administration may be associated with adverse outcomes such as PDA, chronic lung disease, and mortality (65). Volume support can increase preload and hence CO. Hence, in the absence of hypovolemia, volume support of 10–20 ml/kg over 30–60 min may be reasonable. Functional echocardiography can be of assistance in determining volume status and following changes with intervention.

Abnormal vasoregulation is the major contributor to neonatal shock. Vasopressor–inotropes, inotropes, and lusitropes have been extensively used in the management of neonatal shock, surprisingly without robust data directing such management. Dopamine and epinephrine are vasopressor–inotropes and as such, increase both SVR and myocardial contractility. Dobutamine is an inotrope with a variable peripheral vasodilatory action. Milrinone is also an “inodilator” that decreases peripheral vascular resistance but with variable inotropy in newborns due to its age dependent hemodynamic effects (66). Its use has been described with coexistent pulmonary hypertension (67, 68). Phenylephrine and vasopressin are two pure vasopressors and can be of benefit in catecholamine resistant vasodilatory shock. Vasopressin has been recently discussed to have added benefit in the management of hypotension associated with persistent pulmonary hypertension given the pulmonary vasodilatory action mediated *via* V1 receptors, but more data are needed before recommendations can be given for wider use (69).

Commonly used inotropes and vasopressor drugs used in neonatal shock are summarized in Table 3. It is prudent to understand their site of action and hemodynamic effects while managing critically ill infants with hemodynamic instability. The physiological information gained by bedside functional echocardiography may help in making a logical choice of medications depending upon the underlying physiology and the desired hemodynamic effects. For example, patients with shock may warrant use of vasopressor therapy while patients with impaired cardiac function may need more inotropic therapy. Recently, there has been interest in studying the effects of pentoxifylline in neonatal shock, and some studies have shown a positive effect of hemodynamic instability, decrease in hospital stay and mortality especially in infants with gram negative septicemia. However, currently, it is not being routinely used or recommended in clinical practice to improve hemodynamic instability and more studies are required to study its significance (70).

**Table 3:** Commonly used inotropes and vasopressor drugs in neonatal shock.

Name of drug	Dose	Site of action	Hemodynamic effects
Dopamine	1–4 μg/kg/min	Dopaminergic receptors (1 and 2)	Renal and mesenteric dilatation
	4–10 μg/kg/min	α receptors	Inotropic effects
	11–20 μg/kg/min	β receptors	Vasopressor, increase SVR and increase PVR

Dobutamine	5–20 μg/kg/min	β1 and β2 receptors, some effect on α receptors	Inotropic effects; decrease SVR; increase cardiac output

Epinephrine (adrenaline)	0.02–0.3 μg/kg/min	α1 receptors	Inotropic effects; decrease SVR
	0.3–1 μg/kg/min	β1 and β2 receptors	Vasopressor effects; increase SVR

Nor-epinephrine (nor-adrenaline)	0.1–1 μg/kg/min	α1 and α2 receptors	Vasopressor effects; increase SVR

Hydrocortisone	1–2.5 mg/kg; 4–6 hourly	Enhance sensitivity to catecholamines	Uncertain—enhance sensitivity to catecholamines

Vasopressin	0.018–0.12 U/kg/h	Vasopressin 1 receptors	Increase SVR; no inotropic effect

Milrinone	50–75 μg/kg/min bolus followed by 0.25–0.75 μg/kg/min	Phosphodiesterase III inhibitor and produces effects at β1 and β2 receptors	Inodilator effects; lusitropic effects; increase contractility; and decrease SVR

Levosimendan	6–24 μg/kg/min bolus followed by 0.1–0.4 μg/kg/min	Multiple action including Phosphodiesterase inhibitor effect on higher doses	Inodilator effects; increase contractility without increasing myocardial oxygen demand

*SVR, systemic vascular resistance; PVR, pulmonary vascular resistance*.

Beyond the first hour of stabilization, the updated ACCM guidelines emphasize the use of goal directed therapy with additional therapeutic end points, some involving the use of functional echocardiography. Few of the goals mentioned include central venous oxygen saturation of >70%, cardiac index between 3.3 and 6.0 l/min/m^2^, SVC flow >40 ml/kg/min, and ruling out suprasystemic right sided pressures and right ventricular failure on echocardiography (1).

Early goal directed therapy (EGDT) has been well studied in adult population. From a single center study (2001), Rivers et al. reported that EGDT provides significant benefits with respect to outcome in patients with severe sepsis and septic shock (44). Following this study many centers adopted EGDT. However, recent muticenter trials (ProCESS trial, ProMISe trial, and ARISE trial) from North America, United Kingdom, and Australasia failed to show such benefits as compared with local resuscitation protocols (71–73). In fact, they reported an increased use of resources without any improvement in the outcomes of adult patients with septic shock (71–73). In a patient-level meta-analysis, PRISM investigators reported that EGDT did not result in better outcomes than usual care and was associated with higher hospitalization costs across a broad range of patient and hospital characteristics (74).

In contrast with adults, EGDT has not been well studied in neonates and children. The ACCM guidelines (2007) recommended its use in neonatal shock; however, there is not widespread use of EGDT in management of neonatal shock. This is partly due to the fact that septic shock accounts for only a small percentage of shock in the NICU and partly due to the lack of non-invasive measures for hemodynamic monitoring. There is limited evidence on use of central venous oxygenation, cardiac index, non-invasive CO monitoring, and assessing wedge pressure in neonates, and some the parameters are being evaluated in the research studies. However, at this stage, they have limited role in the clinical practice. Modern assessment modalities such as functional echocardiography and NIRS offer non-invasive methods of hemodynamic assessment. Functional echocardiography in particular can be a huge asset to the neonatologist in the initial stabilization and subsequent monitoring in intensive care unit. Various echocardiographic measures are discussed further in the next section.

## Role of Echocardiography in Shock

Functional echocardiography refers to bedside point of care echocardiography that can provide real time hemodynamic information by assessing cardiac function, loading conditions (preload and afterload) and CO (Table 4). It is non-invasive, portable and can give real time analysis of physiological information, which in conjunction with clinical assessment, can help in guiding targeted specific therapy. Various guidelines have been published to standardize the use of functional echocardiography in the NICU (75–78). Its use is especially vital in the intensive care setting where studies have shown that clinical management may change in 30–60% cases in response to echocardiography (79–81). Indeed, expert consensus statement has emphasized the importance of echocardiography in the management of shock (1, 82).

**Table 4:** Bedside focused echocardiography/focused cardiac ultrasound (FoCUS) in neonatal shock.

Fast cardiac ultrasound (FoCUS)/focused echocardiography in shock		

Type of assessment	Echocardiographic assessment	Echocardiographic view(s)
Qualitative assessment of cardiac function and filling	Cardiac filling by “eyeballing” (Figure 3)	Apical 4 chamber view (A4C) and parasternal long axis view (PLAX)

	Assessment of inferior vena cava for collapsibility to assess hypovolemia (Figure 4)	Subcostal view
	Visual assessment of volume overloading (Figure 3)	A4C and PLAX views
	Cardiac function assessment on visualization	A4C and PLAX views
	Cardiac tamponade or pericardial effusion (Figure 5)	Subcostal view, A4C, and PLAX views

Qualitative assessment of pulmonary hypertension	Hypertrophy and/or dilatation of right ventricle Flattening of interventricular septum (Figure 6) Right to left or bidirectional shunt across patent ductus arteriosus Bidirectional shunt across foramen ovale	A4C and PLAX views Parasternal short axis view (PSAX) High left PSAX “ductal” view Subcostal view

“Fast” quantitative assessment of pulmonary hypertension	Assessment of pulmonary artery systolic pressure (PAP) by assessing tricuspid valve regurgitation (Figure 7) Right ventricle to left ventricle (LV) ratio Eccentricity index	A4C view or modified PSAX PSAX view PSAX view

“Fast” quantitative assessment of cardiac function	LV fraction shortening (FS%) Tricuspid annular plane systolic excursion	PLAX view A4C view

### Assessment of Etiology of Shock and Ruling Out CHD and PDA

An initial comprehensive echocardiographic study can aid in ruling out a congenital heart lesion, particularly pulmonary atresia and coarctation of the aorta. In addition to ruling out CHD, this should also include assessment of the PDA with its effects on cardiac hemodynamics. The size of the duct (>1.5 mm at the point of maximum constriction is considered significant), direction of shunting, left atrial to aortic root ratio (LA:Ao ratio, over 1.4 is significant), left pulmonary artery (diastolic velocity, >0.2 m/s is considered significant), and pattern of diastolic flow in the post ductal descending aorta can be used to determine hemodynamic significance of a PDA. Following this, the next step in management is identification of underlying pathophysiology and categorization of shock as distributive, hypovolemic, obstructive, cardiogenic, or dissociative (Table 4).

Below is a brief description of echocardiographic assessment of preload/cardiac filling and cardiac function and evaluation of pulmonary hypertension. The detailed assessment of cardiac function and evaluation of hemodynamics on echocardiography has been published in Frontiers in Pediatrics (83) which is available *via* open access.

### Echocardiographic Assessment of Preload and Fluid Responsiveness

**Figure 3:**
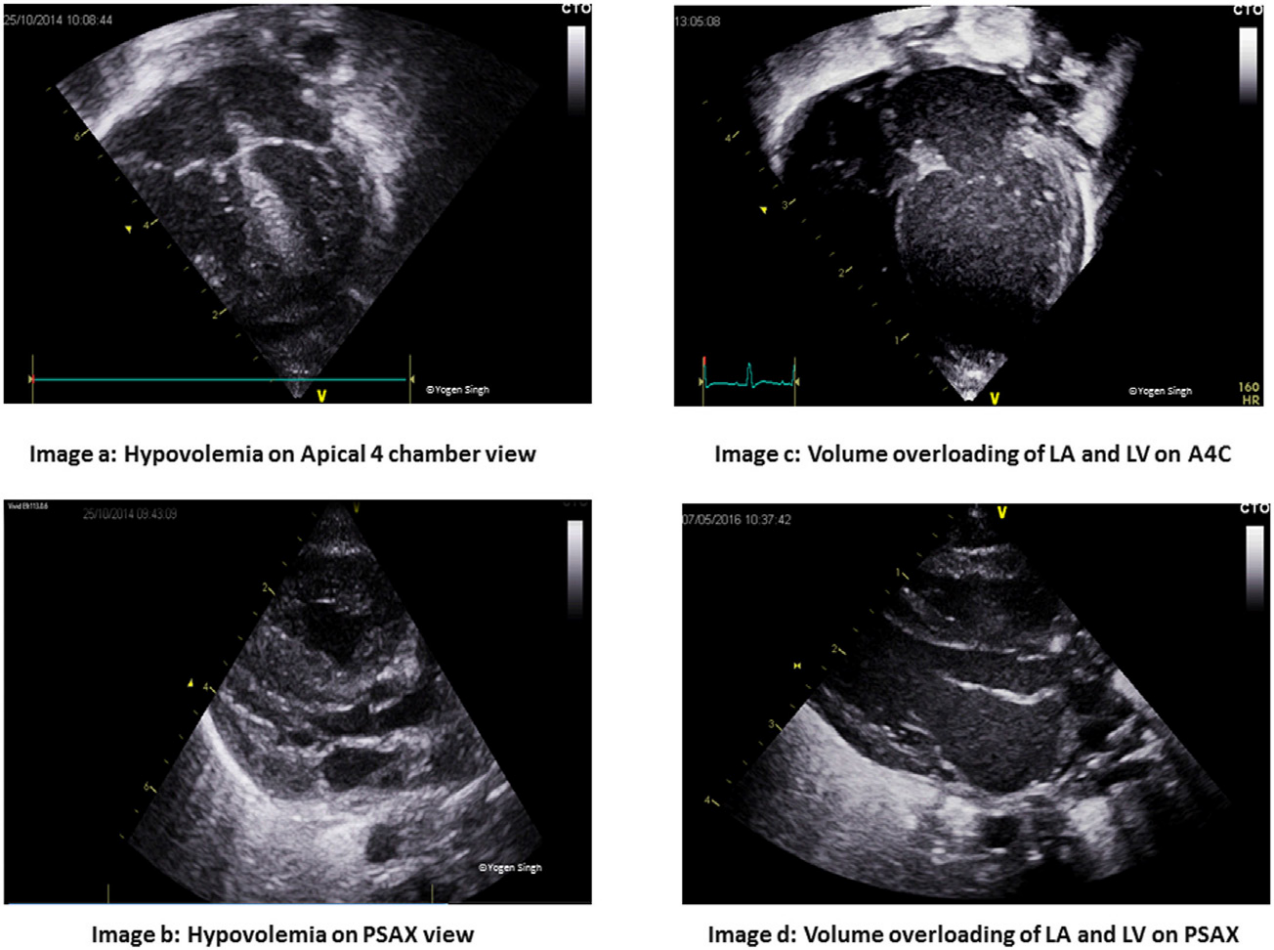
Assessment on cardiac filling on visual inspection “eyeballing.” Images **(A,B)** show under-filled heart in apical 4 chamber (A4C) and parasternal long axis (PLAX) views. Images **(C,D)** show volume overloading of left atrium (LA) and left ventricle (LV) in A4C and PLAX views.

**Figure 4:**
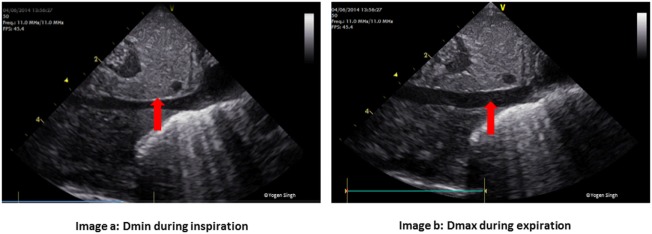
Physiological variation in inferior vena cava (IVC) diameter. Normal collapsibility of **(A)** IVC during inspiration (*D*_min_) and **(B)** expansion during expiration (*D*_max_). In hypovolemia, IVC may be collapsed while in hypervolemia there is minimal or no collapsibility.

Preload assessment of the heart is crucial in management, but it can be affected by multiple factors such as changing lung compliance and presence of mechanical ventilation. Such an assessment can be done by examining the LV, inferior vena cava (IVC), and the right heart. Qualitative assessment includes “eyeballing” the heart in apical four chamber view (Figure 3) while quantitative assessment involves measuring left ventricular volumes and collapsibility index of the IVC (Figure 4).

**Figure 5:**
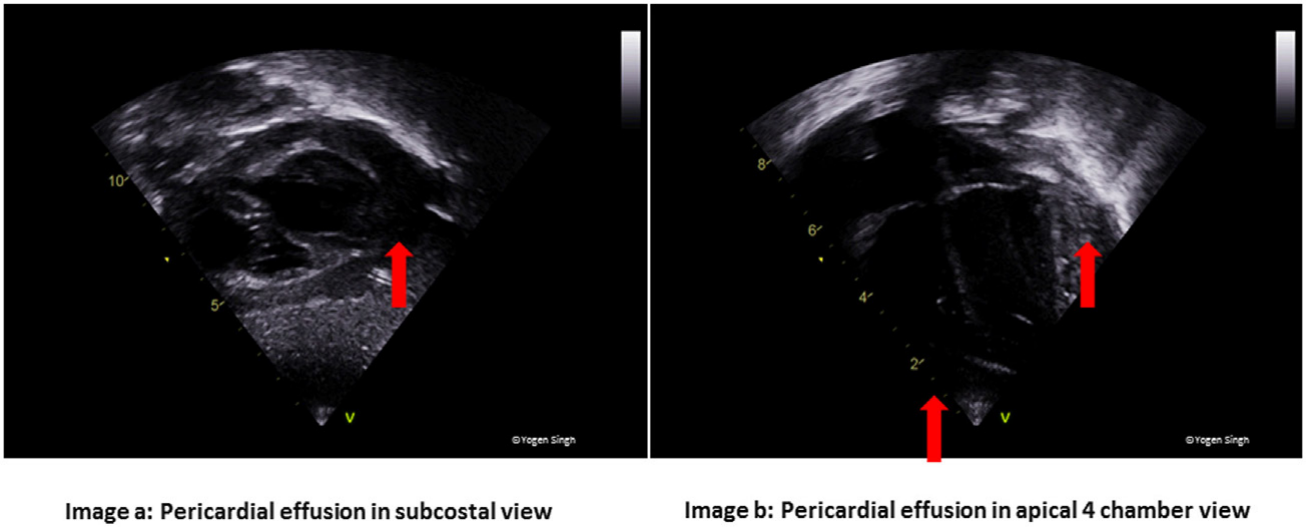
Pericardial effusion in **(A)** subcostal and **(B)** apical 4 chamber views. In large pericardial effusion and cardiac tamponade, there may be collapse of cardiac chambers—first seen collapse of right atrium followed by right ventricle.

Simpson’s biplane method can be used to assess the left ventricular end-diastolic area and volume. Neonates may have physiologic right ventricle (RV) dominance, but in general, the right ventricular dimensions are smaller than those of the LV. A dilated right atrium (RA) may indicate volume overloading of the right side of the heart and in the presence of bowing of intra-atrial septum toward the left atrium may indicate elevated right atrial pressure and hence pulmonary hypertension. By contrast, the triad of a “kissing” small LV cavity, RV size, and a normal or small RA is strongly associated with hypovolemia.

Serial quantitative assessments can be used to monitor “fluid responsiveness,” which can further aid in management. A variation of >15% in left ventricular outflow tract velocity time integral (VTI) during inspiration and expiration has been reported to have high predictive value with a sensitivity and specificity exceeding 90% (84, 85). Similarly, IVC collapsibility index >55%, calculated by measuring the maximum (*D*_max_) and minimum (*D*_min_) IVC diameter from the subcostal view also predicts fluid responsiveness (86). IVC distensibility index exceeding 18% may also be predictive of fluid responsiveness (86).

### Echocardiographic Assessment of LV Function

The qualitative measures include “eyeballing” of the contractility from the apical four chamber view, PLAX, parasternal short axis view (PSAX), or subcostal view (Figure 3). This may be prone to intra- and interobserver variability. Normal LV dimensions in term and preterm infants have been established (87). Quantitative assessments of ventricular function include FS, ejection fraction (EF), Doppler pattern of LV filling (E and A waves at the mitral valve), and tissue Doppler imaging. FS and EF can be obtained using M mode in PLAX or PSAX views or by using Simpson’s method in apical four chamber and apical two chamber views. FS can be affected by regional wall motion abnormalities. EF measurements can be affected by mechanical ventilation, relative tachycardia and non-elliptical LV shape in neonatal ICU. Normal FS in neonates and children is between 26 and 46% (88, 89). Normal EF is >55%, 41–55% is mild reduction, 31–40% is moderate reduction, and 30% is considered marked reduction (79, 90).

The above measures may be affected by load conditions. Appropriate assessment of myocardial activity requires measurement of load independent measures such as relation between velocity of circumferential fiber shortening and LV wall stress indices (24).

Newer more accurate yet less feasible modalities at this time include speckle tracking, strain rate, and 3-D imaging.

### Echocardiographic Assessment of RV Function and Pulmonary Hypertension

**Figure 6:**
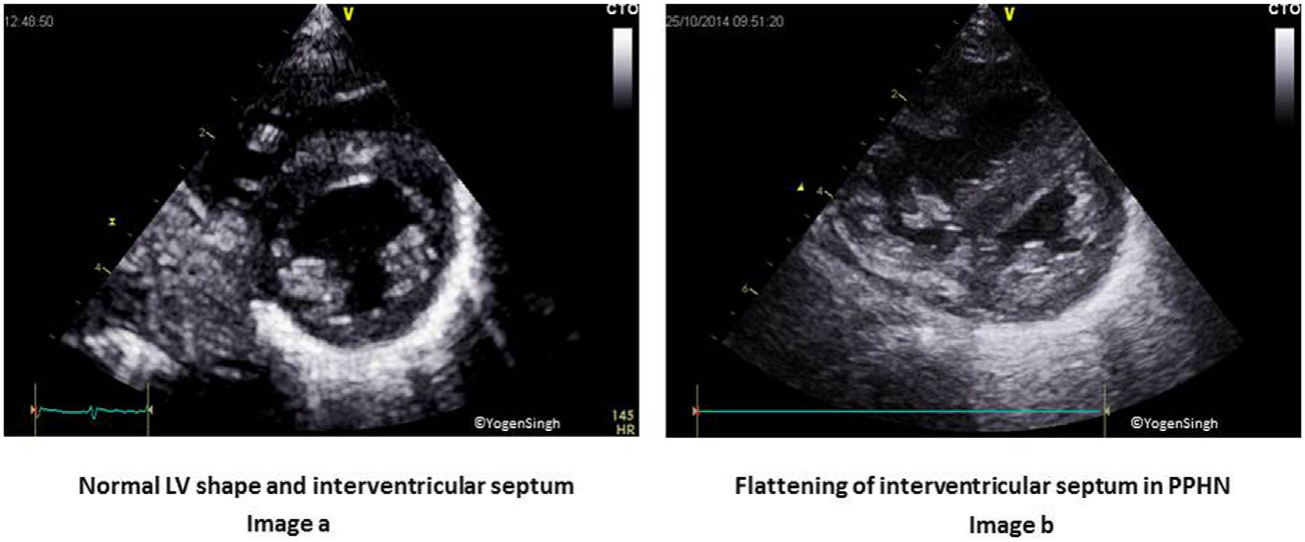
Interventricular septum (IVS) and left ventricle (LV) shape in pulmonary hypertension on visual inspection. Image **(A)** shows normal circular LV and IVS shapes. Image **(B)** shows right ventricular dilatation and hypertrophy of right ventricle, flattening of IVS and “D” shaped LV in pulmonary hypertension.

**Figure 7:**
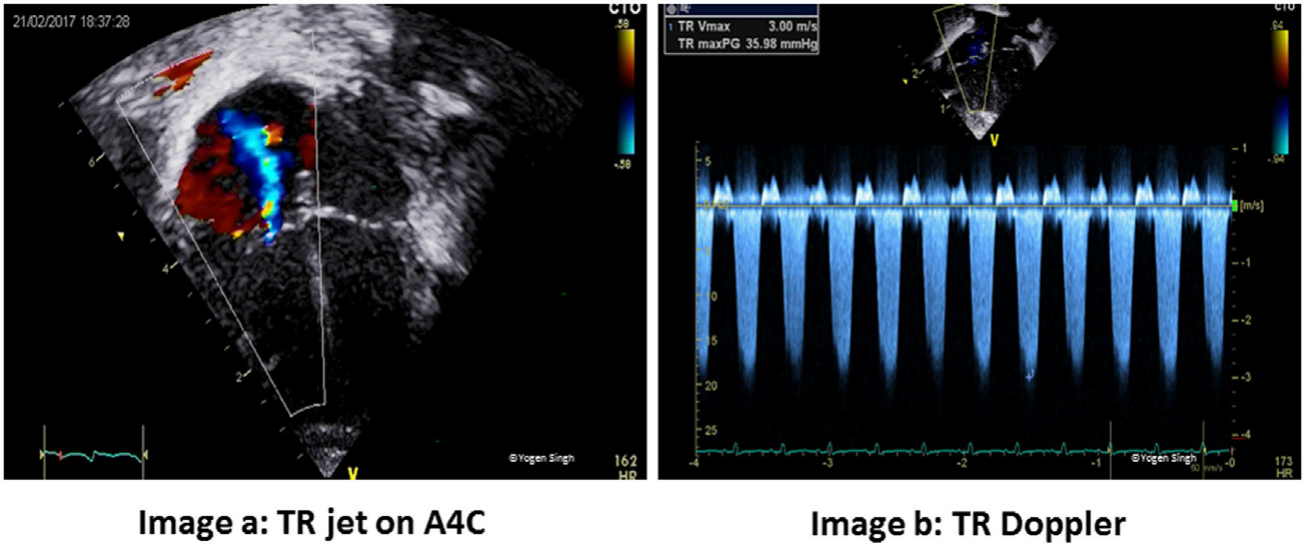
Quantitative assessment of pulmonary artery systolic pressure (PAP) by measuring tricuspid valve regurgitation velocity [tricuspid regurgitation (TR) jet]. PAP = right atrial (RA) pressure + pressure gradient between RA and RV (estimated by TR jet). **(A)** TR jet on A4C. **(B)** TR Doppler.

Persistent pulmonary hypertension of the newborn is a common condition in the NICU. A detailed assessment of RV function is out of scope of this review article and readers may refer to guidelines for assessment of RV function in neonates which have been published in American Journal of Echocardiography (91). Assessment of pulmonary artery pressures in the presence of tricuspid regurgitation (TR) jet (Figure 7), assessment of ductal or atrial shunt with the direction of flow, assessment of interventricular septum and LV shape (Figure 6), tricuspid annular plane systolic excursion, and pulmonary artery acceleration time are some of the measures used in assessment of RV function. Similarly, RV myocardial performance index (MPI) may be used to assess the RV function, and its role in assessing pulmonary hypertension is pivotal in absence of TR. In preterm infants with persistent high PVR have reported to have high MPI values. However, using MPI as a sole marker of global RV function is currently not recommended by the American Society of Echocardiography, and it should be used in conjunction with other parameters (92, 93).

### Echocardiographic Assessment of Systemic Perfusion

This can be assessed through estimation of LV and RV outputs (RVOs) and systemic blood flow measures. RVO and LV output (LVO) can be easily measured using VTI proximal to the pulmonary valve and the AV valve, respectively. The LVO is an estimation of systemic blood flow, and RVO is an estimation of systemic venous return in the absence of cardiac shunts. However, both LV and RVOs may be affected by the presence of fetal shunts. In spite of limitations such as significant intra observer variability, effect of shunts and errors with high angle of insonation, biventricular output measures are commonly used due to reliability with experienced echocardiographer. These values can be trended to follow the impact of interventions in real time. SVC flow may be unaffected by such fetal shunting and has been discussed as a surrogate for CBF but with conflicting reports about association with impaired neurodevelopmental outcomes (4, 32, 34, 36).

## Echocardiographic Prediction of Hypovolemia and Fluid Responsiveness

One of the challenges with using diameters of vessels in newborns is the standardization of the size based on the infant. A ratio between vessels accounts for this. As an assessment of preload to the heart, the IVC is a useful marker for adequate fluid balance. Coupled with the descending aorta in cross section, the IVC/Ao ratio is a useful age/size adjusted marker for assessing a low volume status. Chen et al. demonstrated that an IVC:Ao ratio 0.8 was associated with dehydration (86% sensitivity) in pediatric patients with gastroenteritis. Further work needs to be done in the premature newborn population but the IVC/Ao ratio may have promise as an objective measure of fluid status (94).

## Conclusion

Shock in the newborn period is associated with unique pathophysiologic states that need careful assessment and individualized approach for management. Early recognition of shock and its underlying pathophysiology is critical in instituting early target specific intervention, which may improve outcomes in patients with neonatal shock. A focused bedside functional echocardiography can provide vital anatomic and physiologic information to such management. Widespread use is limited because of its lack of availability, structured training programs for neonatologists and data on clinical outcomes. This modality should be further explored to generate data for therapeutic end points that can be used to standardize and protocolize the management of neonatal shock. We recommend that focused echocardiography in neonatal shock should be regarded as an extension of clinical examination and other traditionally used clinical parameters.

## Author Contributions

YS and FV reviewed the literature and prepared the manuscript. YS provided images and tables for the article. AK edited the article and helped in finalizing the manuscript.

## Conflict of Interest Statement

The authors declare that the research was conducted in the absence of any commercial or financial relationships that could be construed as a potential conflict of interest.
